# A Preliminary Study of Quantitative Ultrasound for Cancer-Risk Assessment of Thyroid Nodules

**DOI:** 10.3389/fendo.2021.627698

**Published:** 2021-05-19

**Authors:** Poorani N. Goundan, Jonathan Mamou, Daniel Rohrbach, Jason Smith, Harshal Patel, Kirk D. Wallace, Ernest J. Feleppa, Stephanie L. Lee

**Affiliations:** ^1^ Section of Endocrinology, Diabetes and Nutrition, Boston Medical Center, Boston University School of Medicine, Boston, MA, United States; ^2^ Lizzi Center for Biomedical Engineering, Riverside Research, New York, NY, United States; ^3^ Verasonics, Inc., Kirkland, WA, United States; ^4^ Department of Biomedical Engineering, Rensselaer Polytechnic Institute, Troy, NY, United States; ^5^ General Electric Research, Niskayuna, NY, United States

**Keywords:** thyroid neoplasm, thyroid nodule, thyroid cancer, thyroid biopsy, quantitative ultrasound

## Abstract

**Background:**

Gray-scale, B-mode ultrasound (US) imaging is part of the standard clinical procedure for evaluating thyroid nodules (TNs). It is limited by its instrument- and operator-dependence and inter-observer variability. In addition, the accepted high-risk B-mode US TN features are more specific for detecting classic papillary thyroid cancer rather than the follicular variant of papillary thyroid cancer or follicular thyroid cancer. Quantitative ultrasound (QUS) is a technique that can non-invasively assess properties of tissue microarchitecture by exploiting information contained in raw ultrasonic radiofrequency (RF) echo signals that is discarded in conventional B-mode imaging. QUS provides quantitative parameter-value estimates that are a function of the properties of US scatterers and microarchitecture of the tissue. The purpose of this preliminary study was to assess the performance of QUS parameters in evaluating benign and malignant thyroid nodules.

**Methods:**

Patients from the Thyroid Health Center at the Boston Medical Center were recruited to participate. B-mode and RF data were acquired and analyzed in 225 TNs (24 malignant and 201 benign) from 208 patients. These data were acquired either before (167 nodules) or after (58 nodules) subjects underwent fine-needle biopsy (FNB). The performance of a combination of QUS parameters (CQP) was assessed and compared with the performance of B-mode risk-stratification systems.

**Results:**

CQP produced an ROC AUC value of 0.857 ± 0.033 compared to a value of 0.887 ± 0.033 (p=0.327) for the American College of Radiology Thyroid Imaging, Reporting and Data System (ACR TI-RADS) and 0.880 ± 0.041 (p=0.367) for the American Thyroid Association (ATA) risk-stratification system. Furthermore, using a CQP threshold of 0.263 would further reduce the number of unnecessary FNBs in 44% of TNs without missing any malignant TNs. When CQP used in combination with ACR TI-RADS, a potential additional reduction of 49 to 66% in unnecessary FNBs was demonstrated.

**Conclusion:**

This preliminary study suggests that QUS may provide a method to classify TNs when used by itself or when combined with a conventional gray-scale US risk-stratification system and can potentially reduce the need to biopsy TNs.

## Introduction

Thyroid nodules (TNs) occur in 50% of the older adult population; however, only about 5% of TNs are malignant (1, 2). Currently, conventional, gray-scale, B-mode, ultrasound (US) combined with fine-needle biopsy (FNB) cytology is the standard of care for evaluating TNs (3–5). To improve the predictive value of B-mode imaging, classification systems such as the American College of Radiology Thyroid Imaging, Reporting and Data System (ACR TI-RADS) and the 2015 American Thyroid Association (ATA) TN risk-stratification system have been developed (3, 6). These systems identify high-risk US features such as hypoechogenicity, taller-than-wide configuration, calcification, irregular margins and abnormal lymph nodes to determine suspicion for malignancy. B-mode imaging is dependent on the quality of the US instrument, selection of the US probe, optimal US-instrument settings, acquisition of appropriate diagnostic images, and the interpretation skills of the ultrasonographer. Differences in image acquisition and observer experience and skills lead to significant inter-observer variability (7).

High-risk B-mode-image features used for risk-stratification are more specific for hypoechoic classic papillary thyroid cancer (cPTC) rather than the follicular variant of papillary thyroid cancer (fvPTC) or follicular thyroid cancer (FTC), which tend to be isoechoic and are not associated with microcalcification. Finally, the sensitivity and specificity of FNB cytology in conjunction with commonly used, costly, and time-consuming molecular genomic and gene-expression testing (e.g., ThyroSeq v3, Afirma GSC) range from 91 to 94% and 68 to 82%, respectively (8, 9).

Quantitative ultrasound (QUS) is a non-invasive method of analyzing microarchitectural tissue properties *in situ* and is a modality that may help to improve non-invasive differentiation of malignant from benign TNs. B-mode images display only the amplitudes of the envelope of the radiofrequency (RF) echo signals reflected back from tissue and discard much of the information that is present in the raw RF echo signal. QUS uses the normalized power spectrum of the raw RF signal, and extracts discarded RF-signal information to obtain parameter-value estimates that are a function of effective scatterer size and effective scatterer acoustic concentration (i.e., the product of the number concentration of the scatterers and the square of their acoustic impedance relative to the acoustic impedance of the surrounding medium) as described in detail for clinicians in prior publications (10–13). While B-mode imaging cannot characterize smaller structures, such as acini, ducts, stromal fibers, capillaries, microfollicles, or papilla, QUS has demonstrated an ability to detect differences in the microarchitecture of benign and malignant tissues. It has been used to distinguish between benign and malignant prostate tissue and metastatic tissue in lymph nodes (14, 15). Small studies in mice and humans have shown the potential of QUS to differentiate between malignant and benign TNs (16, 17). The results of a preliminary study of 53 TNs published by our group showed promising classification performance by QUS parameters either when used alone or in combination with a B-mode-based, risk-stratification system to distinguish benign from malignant TNs (18). A combination of three QUS parameters (spectral intercept, I_0_; effective acoustic concentration, EAC; and Nakagami-statistic, µ) produced a receiver operating characteristics (ROC) area under the curve (AUC) value of 0.93.

This is the first large-scale, single-institution, clinical-use study evaluating the utility of QUS in human subjects for cancer risk-stratification of TNs. The aim of this preliminary study is to determine if QUS US can stratify the risk of malignancy of TNs that is similar to the ACR-TIRAD and ATA Risk Assessment systems for reducing the number of thyroid biopsies while not missing a significant number of malignancies of TNs with definitive cytology or pathology results. Since the QUS and B-mode-US characteristics depend on different aspects of the US RF echo from a TN, we explored the potential of QUS in combination with an existing TN risk-stratification system to determine if the number of biopsies could have been further reduced in this cohort.

## Materials and Methods

### Subject Enrollment and Data Acquisition

The study was approved by the Boston University Medical Center institutional review board. Patients, 18 years and older, were recruited from the Thyroid Health Center at Boston Medical Center if they were scheduled for an FNB of one or more TNs based on the 2015 ATA TN guidelines or at the discretion of the treating provider. Patients were recruited when referred to the research team by a member of the Thyroid Health Center. The gray-scale images archived in the Boston Medical Center picture and archiving and communications system (PACS) and reviewed by an investigator (SLL) with considerable experience in thyroid US to determine the cancer risk according to the ATA risk-stratification system and ACR TI-RADS. A GE LOGIQ-E9 US scanner (GE Healthcare, Chicago, IL) modified for RF-data acquisition was used to acquire conventional gray-scale and QUS data. QUS data were acquired by either of two study investigators (SLL or PNG). Following research US data acquisition, patients underwent an US-guided FNB of one or more TNs per standard of care. The biopsy material was acquired for cytopathology and for additional molecular analysis with ThyroSeq genomic classifier (v2 or v3) (CBLPath, Inc., Rye Brook, NY) if required. A second set of patients who had a prior TN FNB (and had a stable nodule size and US gray-scale characteristics since the FNB) was recruited during a routine follow up visit when the research US data were acquired. TNs were excluded for analysis if a significant cystic region was present or macrocalcification existed superficial to the regions of interest in the TN.

### Data Analysis

The final categorization of the TN was based on FNB cytology and surgical pathology results. A TN was classified as benign if its cytology was unequivocally cancer free (Bethesda II) or surgical pathology showed no evidence of malignancy. If a biopsy specimen had an indeterminate cytology (Bethesda III or IV), it was considered benign if molecular testing was negative or if the positive result was associated with a low risk for cancer (e.g., thyroid stimulating hormone receptor mutation or sodium-iodine symporter overexpression). Molecular testing was not used in TNs with Bethesda V cytology according to the recommendations of the molecular test manufacturers, and surgical-pathology results served as the basis for final categorization. A classification of cancer was determined either by a cytology category of Bethesda VI or by a definitive diagnosis based on surgical pathology. In one subject, a TN was determined to be malignant based on the presence of classic high-risk gray-scale US features and cervical adenopathy. Although the TN was not biopsied, the FNB of the suspicious cervical lymph node was positive for metastatic thyroid cancer.

Each RF data set was manually segmented by an investigator (PNG) for QUS processing by identifying the TN in B-mode images and overlapping rectangular regions of interest (ROI) were applied to ten cross-sectional planes of the nodule. Spectral-parameter estimation techniques have been described in prior publications (14). The backscatter coefficient of each ROI was estimated from the normalized power spectrum. The normalized power spectrum was obtained by dividing the power spectrum of the sample signal by the power spectrum of the RF echo-signal data from a reference phantom at the same depth as the sample data source. Attenuation correction was performed using a nominal attenuation value of 0.5 dB/MHz/cm. The normalized spectrum was fit to linear and Gaussian-form-factor models. In total, five QUS estimates were computed for each ROI, and mean values and standard deviations within TNs were used for classification. Specifically, effective scatterer size (ESS) and effective scatterer concentration (ESC) were obtained from the Gaussian-form-factor model and midband fit and intercept (I_0_) were obtained from the linear model. The fifth QUS estimate was the Nakagami shape parameter (µ), which was computed using a maximum-likelihood estimator to fit the probability density function of the envelope-detected RF signals to the probability density function of the Nakagami distribution.

A linear-discriminant classifier was trained and tested to assess the ability of the QUS parameters to differentiate malignant and benign TNs. Classification performance was expressed using standard ROC methods as the AUC. The ROC AUC values and 95%-confidence intervals (CIs) were obtained using 10-fold cross validation to guarantee that the classifier was not overtrained and able to independently assess previously unseen cases. The mean and standard-deviation values for each QUS parameter were calculated. Initially, the classification performance for each feature was tested alone and then linear combinations of features were tested. To linearly combine the features, the standard Fisher linear discriminant approach was used to test all possible combinations of the five QUS parameter means and standard deviations. Statistical analysis was performed using the MATLAB statistics toolbox (The MathWorks, Inc., Natick, MA).

## Results

309 patients with 333 TNs were recruited for the study. Fourteen TNs were not included in the analysis because the data could not be recovered from the US instrument. Thirty-four TNs were excluded when the signal-processing software detected an error in the data being analyzed and blocked further processing. These data errors occurred randomly, and were not associated with any nodule pathology or sonographic appearance. In five TNs, difficulty existed in identifying the region of interest (ROI) during analysis (e.g., isoechoic nodules with indistinct margins), and in 19 TNs, significant cystic areas or macrocalcifications existed that interfered with the propagation of the US beam and with the analysis and, therefore, these TNs were excluded. Thirty-one TNs were excluded because cytopathology results were insufficient or incomplete at the time of analysis, and three were excluded because non-thyroid pathology was present (i.e., parathyroid adenoma, parathyroid carcinoma, and hyalinizing trabecular neoplasm). Only two non-invasive follicular tumors with papillary like features (NIFTPs) were present with analyzable data confirmed after surgery. NIFTPs were excluded from analysis because it has been considered to be a pre-malignant lesion that cannot be classified as completely benign or malignant. Consequently, the final analysis included 225 TNs from 208 patients. Of these, 167 patients had RF echo-signal data acquired immediately prior to the biopsy being performed. The remaining 58 patients had RF data acquired after an FNB with an interval of 26 days to 85 months. **Table 1** shows the baseline characteristics of the patient cohort and the TNs. Of the 225 TNs, 201 TNs were classified as benign and 24 were classified as malignant. **Table 2** shows the Bethesda classification, molecular analysis, and surgical pathology, if available, for the TNs.

**Table 1:** Basic demographic data and nodule characteristics.

	Total cohort	QUS data acquired prior to FNB	QUS data acquired after FNB
**No. of patients**	208	154	54
**Age (average in years) (p=NS)**	52.8 (SD 14.95)	52.4	54.2
**Gender (p=NS)**			
- **Female (%)**	172 (82.7%)	127 (82.5%)	45 (83.3%)
- **Male (%)**	36 (17.3%)	27 (17.5%)	9 (16.7%)
**No. of nodules**	225	167	58
**Average maximal diameter (cm)**	2.5 (range 0.9 – 7)	2.6 (range 0.9 to 7)	2.3 (range 1 to 6.5)
**Surgical outcome**	34	31	3
- **Benign**	14	13	1
- **Classic PTC**	15	13	2
- **Follicular variant PTC**	3	3	0
- **Follicular thyroid CA**	1	1	0
- **Anaplastic thyroid CA**	1	1	0
Final classification*			
- **Benign**	201	145	56 (p=0.0467)
- **Cancer**	24	22	2

SD, standard deviation; NS, nonsignificant.

*based on cytology, molecular testing and/or surgical pathology.

**Table 2:** Cytology, molecular testing and surgical pathology outcomes for thyroid nodules.

Cytopathology	Total cohort n = 225	Molecular testing performed	Positive molecular testing	Nodules undergoing surgery with surgical pathology results available for review	Surgical pathology results
**Bethesda I**	6 (2.6%)	3	3 [HRAS (1), EIF1AX(1), PAX8-PPARG(1)]	6	Benign – 3 cPTC – 2 fvPTC – 1
**Bethesda II**	144 (64%)	1	0	1	Benign – 1
**Bethesda III**	49 (21.8%)	48	13 [BRAF v600E(1), NRAS(2), HRAS(2), KRAS(2), EIF1AX(1), EZH1(1), NIS overexpression (1), TSHR mutation (2), PAX8-PPARG(1)	12	Benign – 10 cPTC-1 fvPTC – 1
**Bethesda IV**	9 (4%)	9	3 [HRAS(1), EIF1AX (1), TSHR mutation (1)]	2	fvPTC – 1 FTC-1
**Bethesda V**	4 (1.8%)	4	4 [BRAF V600E (2), HRAS (1), TERT (1)]	4	cPTC – 3 Anaplastic – 1
**Bethesda VI**	12 (5.3%)	0	0	9	cPTC – 9
**No FNA performed for nodule**	1 (0.004%)*	0	0	0	0

cPTC, classic papillary thyroid cancer.

fvPTC, follicular variant of papillary thyroid cancer.

*Nodule has associated abnormal cervical lymph node that was positive for malignancy on FNA.

### Performance of QUS

**Figure 1:**
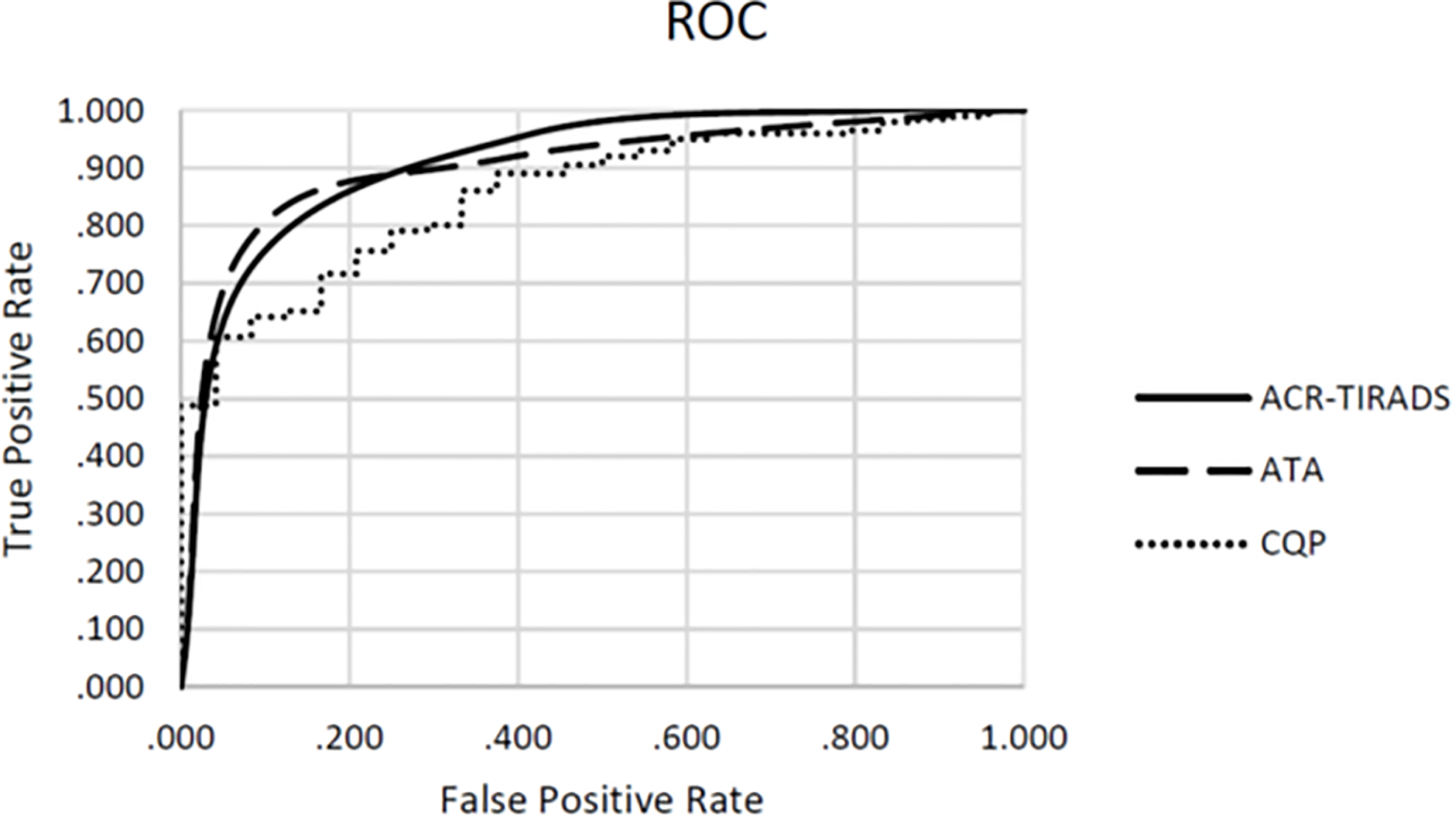
Receiver operating characteristics (ROC) for combination QUS parameters (CQP), ATA risk-stratification and ACR TI-RADS.

**Figure 2:**
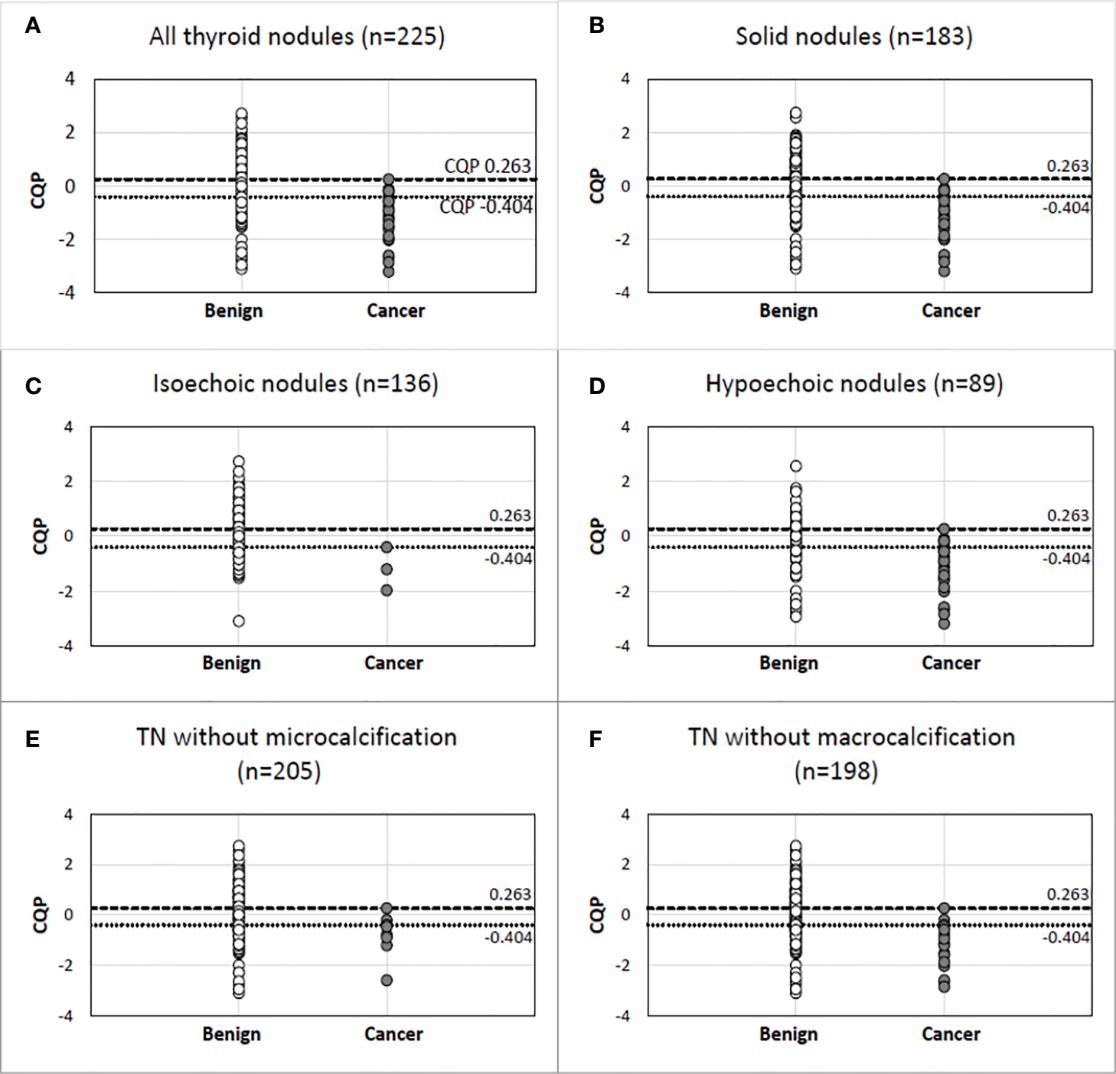
Combination QUS parameter (CQP) distribution in malignant and benign pathology in all thyroid nodules **(A)** and in nodules selected based on specific gray-scale ultrasound features **(B–F)**. Benign nodule (open circle). Cancer (closed circle).

**Table 3** shows the performance of individual QUS parameters. The best single-parameter performances were produced by I_0_ with a ROC AUC value of 0.742 +/- 0.046 and ESC with an AUC value of 0.702 +/- 0.049 as shown. The normalized standard deviation of selected QUS parameters showed improved performance with an AUC of 0.769 +/- 0.047 and 0.72 +/- 0.064 for I_0_ and ESC respectively. Linear discriminant analyses revealed that the optimal linear combination of QUS parameters (CQP) was [(0.143 x ESC) + (0.018 x ESS) + (3.198 x µ) - (0.391 x σESC) - (14.896 x σESS/mean of ESS) - 3.062], which produced an AUC value of 0.857 +/- 0.033 as shown in **Figure 1**. **Figure 2** illustrates the performance of CQP for all TNs and for TNs selected based on individual TN characteristics, e.g., solid, echogenicity and exclusion of calcification. While CQP values overlap for benign and cancerous TNs, a distinct clustering of CQP values occurs for malignant TNs that allows defining thresholds for ruling out cancer.

**Table 3:** QUS parameter performance.

Parameter	AUC (± CI)
Intercept (I_0_)	0.742 ± 0.046
Effective scatterer concentration (ESC)	0.702 ± 0.049
Midband fit (MF)	0.695 ± 0.048
Nakagami shape (µ)	0.637 ± 0.063
Effective scatterer size (ESS)	0.584 ± 0.058

### Comparison of QUS Performance and Gray-Scale Risk-Stratification Systems

We examined the utility of QUS compared with ACR TI-RADS and the ATA nodule classification systems to reduce FNBs of benign TNs. When applied by an experienced thyroid sonographer the ACR TI-RADS system produced an AUC value of 0.887 +/- 0.033 and the ATA system produced an AUC value of 0.880 +/- 0.041. There were no statistically significant differences when comparing these values with the ROC performance of CQP (p=0.327 and p=0.367 respectively). In this cohort of TNs that have been selected for biopsy by the treating providers, ACR TI-RADS would not have recommended a biopsy in 89 (39.6%) TNs, with a single false-negative determination. The ATA system would have prevented 14 biopsies (6.2%) with no false-negative determinations. The ATA system is used by our endocrine clinicians to determine the need for FNB, and hence there was a lower number of TNs excluded for biopsy when reviewed by investigators. In comparison, if a CQP value of 0.263 or less was used to recommend tissue sampling, while providing a zero percent false negative rate, a biopsy would be avoided in 98 TNs (43.6%) (**Table 4**). Similarly, using a threshold of -0.404 to provide a false negative rate of less than 3% (which is currently considered to be the acceptable false-negative FNB rate for TNs with Bethesda II or indeterminate TNs with negative molecular testing), 148 (65.8%) TNs would not undergo a biopsy. The sensitivity, specificity, positive predictive value, and negative predictive value for ACR TI-RADS, ATA system and the CQP are provided in **Table 5**.

**Table 4:** Number of nodules excluded from biopsy of the 225 TN cohort based on ACR-TIRADS using recommended size threshold vs in combination with QUS parameter (CQP) cutoffs.

Classification System	Threshold for avoiding biopsy		
	Traditional ACR TI-RADS criteria**	CQP > 0.263	CQP > -0.404
**ACR TI-RADS [total no. (%)]**	**No. of TNs excluded from FNB [No. of missed cancers]**		
**TI-RADS 1 [6 (2.6%)]**	6 [0]	1 [0]	2 [0]
**TI-RADS 2 [30 (13.3%)]**	30 [0]	14 [0]	19 [0]
**TI-RADS 3 [83 (36.9%)]**	40 [0]	41 [0]	63 [0]
**TI-RADS 4 [74 (32.9%)]**	12 [1]	33 [0]	50 [2]
**TI-RADS 5 [32 (14.2%)]**	1 [0]	9 [0]	14 [2]
	**No. of TNs excluded from FNB (%) [No. of missed cancers]**		
**QUS alone (CQP)**	NA	98 (43.6%) [0]	148 (65.8%) [4]
QUS (CQP) + TI-RADS*	NA	110 (48.9%) [0]	149 (66.2%) [2]

*TN with TI-RADS 1 and 2 are not biopsied, TI-RADS 5 are always biopsied and TI-RADS 3 and 4 are biopsied only if < CQP threshold indicated.

**Nodule size as threshold for biopsy.

**Table 5:** Sensitivity, specificity, positive predictive value (PPV) and negative predictive value (NPV) of ACR TI-RADS, ATA system and CQP parameter.

	Sensitivity (%)	Specificity (%)	PPV (%)	NPV (%)
**ACR TI-RADS**	95	43	16.9	98.9
**ATA system**	100	6.9	11.3	100
**CQP (threshold > 0.263)**	100	48	18.9	100*
**CQP (threshold > -0.404)**	83	71	26	97.3*

*CQP thresholds 0.263 and -0.404 selected to produce a NPV of 100% and 97% respectively.

**Figure 3:**
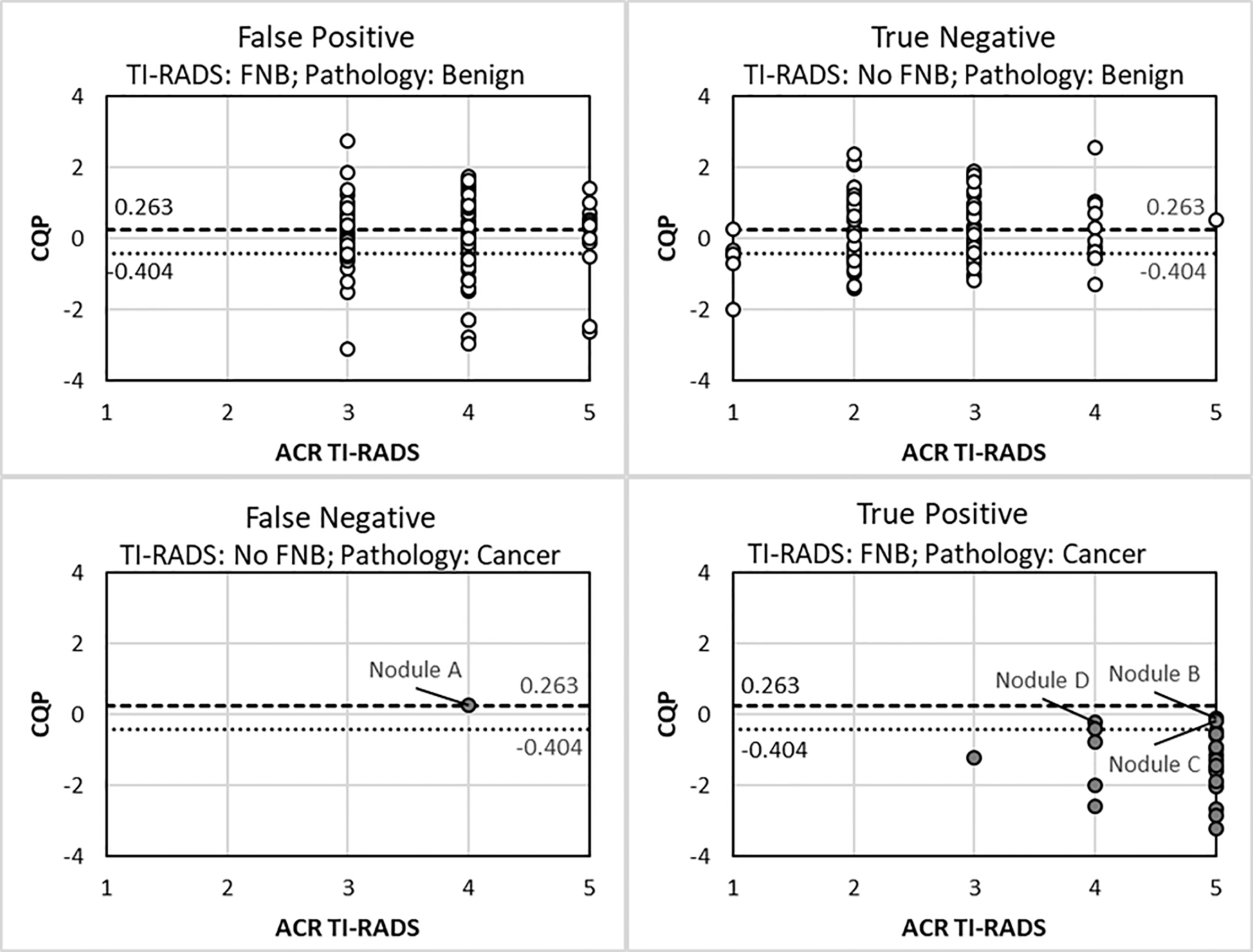
Scatter plot of ACR TI-RADS and combination QUS parameter (CQP) for different FNA decision outcomes. Benign nodule (open circle). Cancer (closed circle).

The single missed cancer when applying the traditional ACR TI-RADS criteria to the cohort was nodule A (Bethesda III cytology with NRAS mutation), which was a low-risk, 1-cm, encapsulated fvPTC with minimal focal capsular invasion without lymphovascular invasion, extra-thyroidal extension, or abnormal lymph nodes on surgical pathology as shown in **Figure 3**, False Negative Panel. In contrast, when using a CQP threshold of 0.263, no false-negative determinations occurred. In addition to nodule A, when using a CQP threshold of -0.404, the three other cancers missed were nodule B (Bethesda IV cytology with positive HRAS mutation, which was a 1.1 cm fvPTC without lymphovascular invasion, extrathyroidal extension or abnormal lymph nodes on surgical pathology), nodule C (Bethesda VI cytology; at the time of writing this manuscript, the patient has not undergone surgery) and nodule D (Bethesda IV cytology with a EIF1AX mutation, which was a 4.5 cm oncocytic variant of FTC with focal vascular invasion on surgical pathology) as shown in **Figure 3**, True Positive Panel. The two patients with a fvPTC only required a lobectomy. These two patients did not follow up at our institution and long-term follow up data are not available. The patient with FTC was treated with radioactive iodine therapy and shows no evidence of persistent disease (ATA current risk: low, and ATA excellent response to therapy) 26 months following the initial thyroid surgery.

### QUS in Combination With Existing Risk-Stratification Systems

The use of a B-mode, risk-stratification systems in combination with QUS was explored to determine if additional FNBs or molecular testing could be reduced as summarized in **Table 4**. If all TI-RADS (TR)-1 and -2 TNs were excluded from biopsy, all TR-5 TNs were biopsied regardless of size and if TR-3 and -4 TNs were biopsied if the CQP was equal to or less than a threshold of 0.263, then 110 of the 225 TNs (48.9%) would not be biopsied without missing a malignancy. Using the same criteria, if a CQP threshold of -0.404 was used, then 149 TNs (66.2%) would not require a biopsy and two malignant TNs would be missed, as shown in **Figure 3**.

## Discussion

TNs are a common occurrence in the adult population. The ACR-TIRAD and ATA Risk Assessment systems based on high-risk sonographic characteristics have very high negative predictive value (NPV) but low positive predictive value (PPV) resulting in unnecessary biopsy of benign nodules. Even when combined with fine needle biopsy, 20-30% of biopsies will have an indeterminate cytology that results in expensive molecular marker testing or surgery for pathological diagnosis. We anticipate that QUS, an US technology that allows a noninvasive method of investigating microarchitectural structures that cannot be assessed by conventional B-mode imaging, will provide additional information to improve the discrimination between benign and malignant thyroid nodules to reduce the number of biopsies of benign nodules and reduce the molecular marker testing and surgery of nodules with an indeterminate (Bethesda III, IV) cytology.

The study cohort had a Bethesda cytology category distribution that is typical of this institution including the indeterminate rate (Bethesda III and IV) of 26%. The overall cancer prevalence was 10.7% in nodules that were selected for biopsy and is also typical of our academic tertiary care referral center. The cancer prevalence is higher in this study cohort compared to the 5% prevalence in all TNs as some nodules are not biopsied because of small size or benign sonographic characteristics (completely cystic or spongiform). The study cohort in which research data were acquired after the FNB was completed had a higher proportion of Bethesda II cytology (76%) as they did not have surgery or refused surgery based on the prior FNB results.

The ACR-TIRADS and the ATA systems are used to determine if a TN can be excluded from biopsy based on size and the absence of high-risk sonographic features. The AUC for ACR TI-RADS and the ATA system to identify cancer in prior published data has a wide range of values between 0.76 to 0.88 and 0.77 to 0.89, respectively (19–22). In this study, the gray-scale US images were reviewed by an endocrine ultrasonographer with more than 25 years of experience, which likely accounts for the excellent performance of the ACR TI-RADS and ATA system in the upper range of published data (0.887 and 0.880, respectively), but may not reflect the results of a clinician in general endocrine practice. Conventional gray-scale US is operator and instrument dependent with a high inter-observer variability, especially for certain features such as the TN margin, volume, and presence of microcalcification (7, 23, 24). This inter-observer variability affects the results of the ATA and ACR TI-RADS classification systems (25–27). By itself, using CQP in this preliminary study, the number of biopsies could be reduced by 44-66%, which equaled or outperformed the ACR TI-RADS as well as the ATA system applied by an expert thyroid sonographer. A further reduction in biopsies was seen with no missed cancers when QUS (CQP>0.263) was used with TIRADS (48.9%) compared to QUS alone (43.6%) suggesting additional studies are necessary to explore the utility of combining modalities to reduce biopsies.

There were several limitations and aspects of QUS application in TN assessment that were not evaluated in this study. Factors affecting the reproducibility of results that are operator-dependent, such as defining the ROI, were not addressed in the current study. Given limited numbers, individual cancer subtypes were analyzed together rather than separately. NIFTPs, which currently are considered a pre-malignant lesion and are a clinically relevant subtype of thyroid tumors, were not included in the current study because of the low prevalence in this cohort of TNs and uncertainty whether to consider this a benign or malignant in the classification for this study. Although NIFTP are considered a benign lesion, resection is recommended because of the potential of future malignant behavior. Since the recommendation is for surgical removal, it would be an error to categorize it as benign requiring no further characterization in the analysis of this data. Identifying isoechoic cancers has traditionally been difficult due to the lack of specificity of this B-mode feature and the frequent absence of an association with other high-risk US features. While QUS appeared to show an encouraging degree of separation between malignant and benign isoechoic TNs (**Figure 2**), only three isoechoic malignant nodules were present in the cohort, which prevents any definitive conclusion. Another subgroup that should be analyzed in the future are autonomously functioning TNs. As described in the results, several TNs were excluded from the final analysis. Other than the presence of significant macrocalcification and cystic areas, the reasons for exclusion are not intrinsic limitations of the imaging method. We intentionally did not include TNs with significant macrocalcification and anteriorly located cystic areas that interfered with US RF propagation through the TN. However, this is a potential limitation for all US technology including B-mode classification systems or elastography because of the artifacts that develop as a result of lower signal attenuation in liquids and strong reflections from macrocalcifications. Finally, reference standards for benign and malignant diagnoses were based on cytology and molecular results in addition to surgical pathology and this may cause false-negative or false-positive results.

The cohort included a total of 225 nodules, with US data being collected either before or after FNB completion, as this was a preliminary study. The 33 patients in our pilot study (ROC AUC 0.93) are a subset of the nodules included in this study (18). With a larger training set in this investigation, the current study produced a different classifier with the optimal performance (ROC AUC 0.857) indicating the need to further investigate larger cohorts of patients to allow the use of separate training and test sets and application of techniques such as machine learning to determine optimal classifiers.

Future prospective studies are required to validate the findings from the current study and further explore the role of QUS in the evaluation of TNs to reduce biopsies of benign TN, possibly reduce the use of expensive molecular testing and reduce unnecessary surgery of benign TNs with indeterminate cytology (Bethesda III, IV). The currently available molecular testing for FNB are “rule out” tests with low positive predictive values because of overlapping molecular findings in benign and malignant nodules. The results of this initial study justify a larger prospective study examining the benefits of combining QUS with other US risk-stratification methods, such as TI-RADS, to further enhance TN-classification.

## Data Availability Statement

The raw data supporting the conclusions of this article will be made available by the authors, without undue reservation.

## Ethics Statement

The studies involving human participants were reviewed and approved by Boston University Medical Campus and Boston Medical Center. The patients/participants provided their written informed consent to participate in this study.

## Author Contributions

PG, JM, DR, KW, EF, and SL conceived and planned the study design. PG and SL collected data and all authors contributed towards processing and analyzing data. All authors contributed to the article and approved the submitted version.

## Funding

The research reported in this publication was supported by the National Institutes of Health grant R21 CA212744 awarded to the Boston Medical Center, SL, Principal Investigator.

## Conflict of Interest

Author DR is an employee of Versonics, Inc. and KW is an employee of the General Electric Research Center.

The remaining authors declare that the research was conducted in the absence of any commercial or financial relationships that could be construed as a potential conflict of interest.
